# Falls in Older Patients with Cancer Undergoing Surgery: Prevalence and Association with Geriatric Syndromes and Levels of Disability Assessed in Preoperative Evaluation

**DOI:** 10.1155/2018/5713285

**Published:** 2018-05-15

**Authors:** Somayeh Fahimnia, Hadi Mirhedayati Roudsari, John Doucette, Armin Shahrokni

**Affiliations:** ^1^Department of Environmental Medicine and Public Health, Icahn School of Medicine at Mount Sinai, New York, NY, USA; ^2^Department of Geriatrics Service, Memorial Sloan Kettering Cancer Center, New York, NY, USA

## Abstract

Falls are common among older adults. However, not much is known about the prevalence of falls among older patients with cancer. In 2015, older patients with cancer referred to Geriatrics service for preoperative evaluation were assessed for fall history, basic and instrumental activities of daily living (ADL and IADL), KPS, and use of assistive device. Of 806 patients, 215 (26.7%) patients reported fall. Incidence of last fall inside and outside home was 54.4% and 45.5%, respectively. Among patients with no falls, 33.6% had KPS ≤ 80 compared to 59.6% with one-time fall and 60.7% with multiple falls (*p* < 0.001). Among IADL, 8.5% of patients with no falls were unable to do shopping compared with 14.7% in one-time fall and 18.8% in multiple fallers (*p* < 0.001). In ADL items, the percentage of patients who were limited a lot in walking outside was 10.7% in no falls, 20.2% in one-time fall, and 27.1% in multiple fallers groups (*p* < 0.001). Only 17.8% of patients with no falls were using canes while 27.7% of patients with one-time fall and 38.8% with multiple falls were using canes (*p* < 0.001). Falls are prevalent among older patients with cancer. Fall history and number of falls are associated with functional status.

## 1. Introduction

Cancer is most prevalent among adults older than 65, an increasingly growing segment of the population. A rise from 61% to 70% in the percentage of all cancers diagnosed among older adults is predicted from 2010 to 2030 [1]. An increasing proportion of older patients with cancer undergo surgery nowadays since cancer resection is no longer limited or denied merely based on advanced age [2]. Older patients considering surgery should undergo a preoperative assessment that includes an evaluation of comorbidities and geriatric conditions [3]. Geriatric syndromes such as falls have been shown to be of predictive value for postoperative outcomes. In a study on general population older adults, it was shown that postoperative outcomes including surgery complications, discharge to a care facility, and early readmission were more common in older patients with recent fall history [4]. Previously it has been shown that in older patients with cancer undergoing surgery, fall history is associated with a higher chance of developing delirium and worse outcomes after surgery [5].

Falls, with an annual incidence of more than one-third among community-dwelling older adults, are considered a serious public health issue [6]. Nonfatal fall consequences in the older adults range from minor lesions and bruises to serious fractures and traumatic brain injuries (TBI) [7]. Up to 90% of hip fractures are caused by falls and the most common cause of TBI among older adults is falls [7]. Additionally, falls in older adults lead to fear of falling which causes further decline in physical and mental abilities, increased risk of falling, and decreasing health-related quality of life [8]. Due to additional risk factors caused by their cancer, older adults with cancer are even at higher risk for falling [9].

Despite its high prevalence and preventive value, falls seem to be overlooked in our assessments for older patients with cancer. Only 10% of appropriate medical record documentation has been reported for geriatric cancer patients who self-reported recent falling [10]. Fall history before cancer surgery can be linked to many aspects of patients' lives such as their disability and dependency level, and the type of care they would need after surgery [11]. This is a study to determine the prevalence of falling among older patients with cancer undergoing surgery and understanding its associations with other geriatric syndromes and functional status in these patients. We also aimed to evaluate the association of activities of daily living and instrumental activities of daily living with fall history and number of falls in older patients with cancer in preoperative setting and assess the use of walking assistive devices among these patients as a preventive measure for falls.

## 2. Methods

This is a retrospective study of cancer patients (age 75 or older) who presented to Memorial Sloan Kettering Cancer Center-Geriatrics Service in 2015 for preoperative evaluation. All patients received comprehensive geriatric assessment (GA) as a part of their preoperative evaluation. Falls were assessed by asking patients about their history of falling in the past 12 months, the number of falls (one or multiple falls), and the context of the last fall. Functional domains of geriatrics assessment included basic and instrumental activities of daily living (ADL and IADL), patient-reported Karnofsky performance status (KPS), timed up and go (TUG), and use of assistive devices (cane, walker, or wheelchair). The preoperative GA was performed by geriatricians, geriatric nurse practitioners, or trained registered nurses in the geriatric service. ADL is a scale to measure the patients' functional status and daily self-care activities. It can be further categorized into basic and instrumental ADL (IADL) [12]. Basic ADL is defined as capability of basic actions necessary for living at home including personal hygiene, mobility, and eating, while IADL refers to more complex tasks than basic activities for living in a community comprising ability to manage finance, drive, use transportation, cook, shop, do laundry, be responsible for one's own medications, and maintain the house [12]. The TUG test is a simple mobility examination to assess the risk of falls in elder patients. It is defined as the number of seconds that takes a person to rise from an armed chair, walk for three meters, turn, walk back to the chair, and sit down [13]. In the present study we used the three cut-off scores of <10 seconds, 10–19 seconds, and >20 seconds [14]. Sociodemographic and clinical characteristics were also retrieved.

## 3. Data Analysis

The chi-square test was used to assess the association between history of falls in the past year and ADL and IADL dependencies. In addition, chi-square was used to assess the association between falls and the use of assistive devices. Microsoft Excel was used for data entry and data analysis was performed using IBM SPSS version 22.

## 4. Results

The study population consisted of 816 preoperative older patients with cancer. The median age among this population was 80 (77, 83) years old. A summary of the patients' sociodemographic characteristics, stratified by having or not having a fall during the past year, is shown in Table 1. Fall history data was available for 806 patients of which 215 (26.7%) reported at least one fall in the past 12 months. Of the 215 patients with positive fall history, 130 (60.5%) reported only one fall and 85 (39.5%) had multiple falls. Falls were more common among patients 80 years or older, females, nonmarried patients, and those who did not live alone. However, the only difference in frequency of falling which achieved statistical significance was between males and females (*p* = 0.025). A total of 117 (54.4%) patients reported that their last fall happened inside the home and 98 (45.5%) said that it occurred outside.

The most common cancer surgeries based on site included urological (25.8%), head and neck (13.4%), colorectal (12%), hepatopancreatic (8.7%), and gynecological (8.3%). Moreover, 392 (48.0%) patients were undergoing ambulatory/minor cancer surgery while 424 (52.0%) needed hospitalization for their surgery.

The patient-reported KPS scores were stratified into two strata, ≤80 and 90–100 (Figure 3). In no fall group, 66.5% of patients had KPS score 90–100 as compared to 33.5% of patients with KPS score ≤80 (*p* < 0.001). Moreover, the percentage of patients with KPS score ≤80 was significantly higher than those with KPS score 90–100 among patients with one-time fall (57.4% versus 42.6%) and multiple falls (61.2% versus 38.8%) (*p* < 0.001 for all).

Timed up and go (TUG) test results were also differentiated as less than 10 seconds, 10 to 19 seconds, and more than 20 seconds (Figure 4). TUG among patients with no fall and one fall and those with multiple falls is shown in Figure 2. TUG test results were significantly associated with number of falls (*p* < 0.001). No fall group had the highest proportion (66.7%) of individuals with TUG < 10 seconds as compared with one-time fall (50.4%) and multiple fall (39.4%) groups (*p* < 0.001). In addition, the percentage of patients with TUG 10–19 and >20 seconds was significantly associated with number of falls (28.2% and 21.4% in one-time fall versus. 32.4% and 28.2% in multiple falls group; *p* < 0.001).

**Figure 1:**
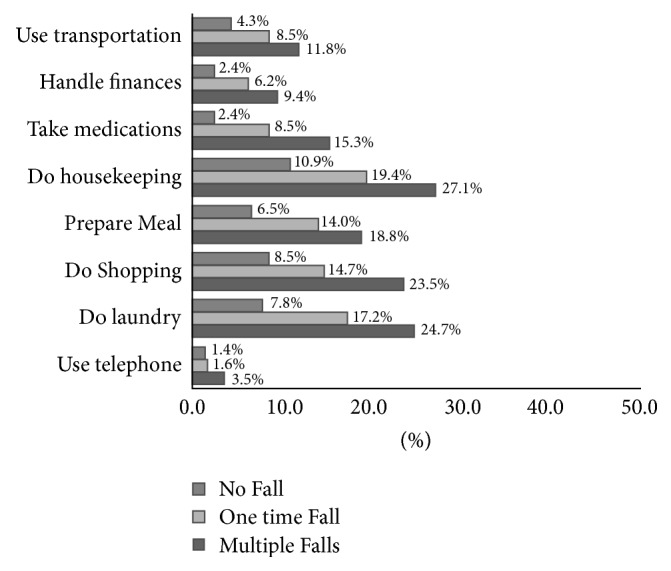
Percentage of patients who had no fall, one fall, and >1 fall who were “unable to do” in different IADL.

**Figure 2:**
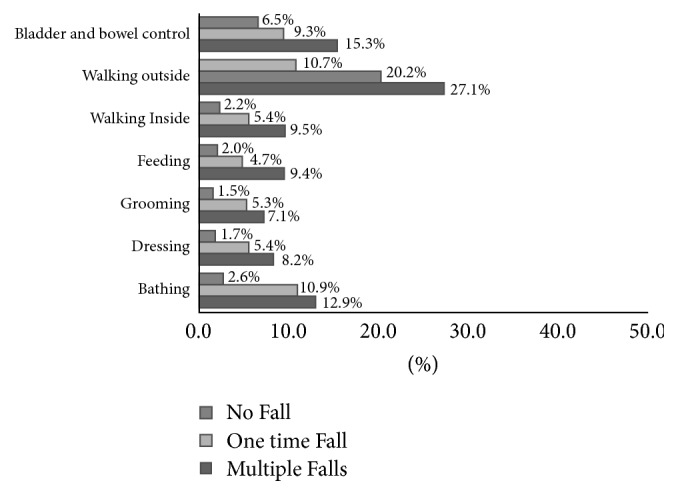
Percentage of patients who had no fall, one fall, and >1 fall who were “limited a lot” in different ADL.

**Figure 3:**
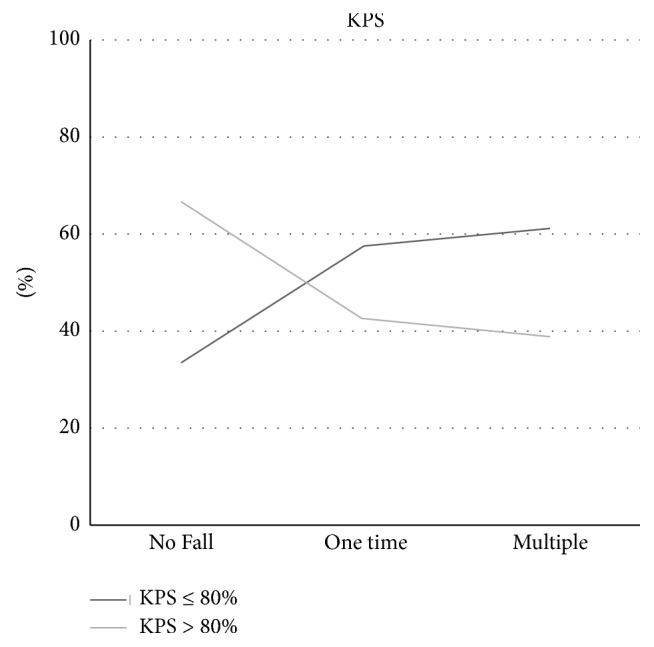
Patient-reported KPS score for fall number groups.

**Figure 4:**
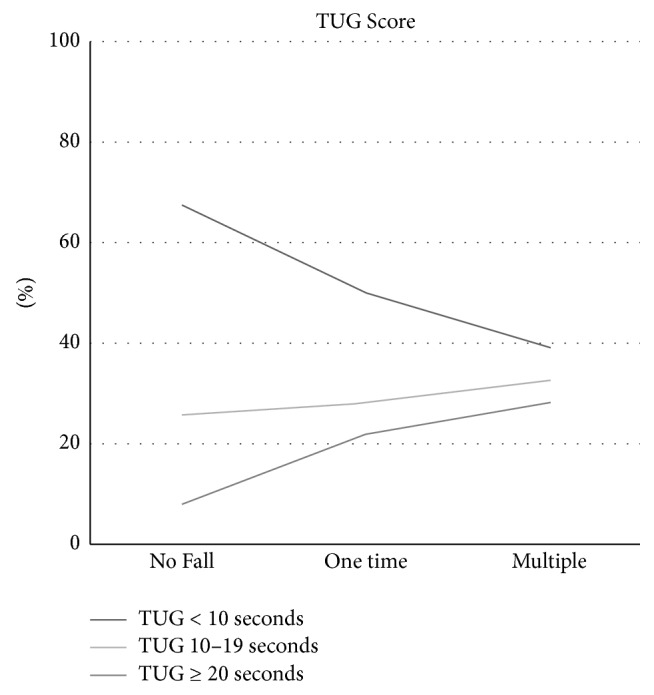
TUG test results for fall number groups.

There was a significant association between patients' level of physical activity and fall numbers for all IADL/ADL questions. There were three levels of function that could be answered by patients for each IADL/ADL question including “not limited at all,” “limited a lot,” and “limited a little” in our questionnaire [15]. In order to show the magnitude of disability associated with fall numbers, we dichotomized each IADL and ADL as the worst category versus all others, specifically, “unable to do” for IADL and “limited a lot” for ADL. The association of IADL and ADL items with fall frequency is shown in Figures 1 and 2, respectively.

The proportion of patients who answered “unable to do” shopping was 8.5% in no fall group, 14.7% in one-time fallers, and 18.8% in those with multiple falls. The percent of patients unable to do light housekeeping activity rose from 10.9% in no fall group to 19.4% in one-time fallers and, 27.1% in those with multiple falls.

Among ADL questions, the percentage of patients who answered that they were limited a lot in walking outside was 10.7% in no fall group, 20.2% in one-time fallers, and 27.1% in those with multiple falls. Furthermore, while only 2.6% of patients in no fall group were very limited in bathing activity, this percentage was reported higher in one-time fall and multiple fall groups (9.3% and 15.3%, respectively). While only 6.5% of patients with no fall history were very limited in bladder/bowel control, this percentage increased to 9.3% and 15.3% in one-time and multiple time fallers, respectively.

**Figure 5:**
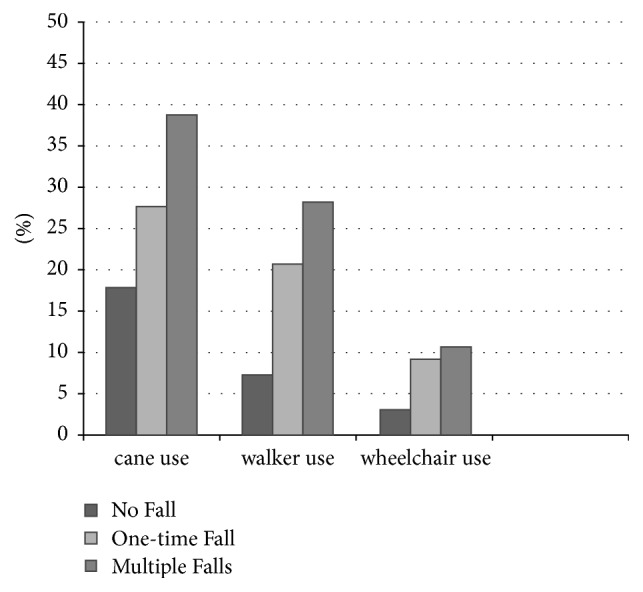
Walking assistive devices usage by each fall number group.

The use of walking assistive devices was also analyzed. 602 (73.8%) patients were not using any devices, 138 (16.9%) used one assistive device, 60 (7.4%) had two devices, and 16 (2.0%) used three devices. The types of assistive devices and their usage or nonusage in each fall frequency group are shown in Figure 5.

## 5. Discussion

We found that at least one out of four (26.7%) older patients with cancer presenting for preoperative evaluation has experienced one or more falls in the past year. This prevalence is consistent with other studies conducted on older adults with cancer [9]. Furthermore, in a recent systematic review about falls in older adults with cancer the prevalence of falls was calculated between 20 and 30% over 3–12 months periods [9].

Within our cohort, 15.9% have experienced one fall, while 9.54% of the cohort experienced more than one fall in the year prior to the preoperative evaluation. This finding is also in line with a prior study that showed 12% and 9% of older patients with cancer having experienced one and more than one fall in the last six months, respectively [16]. Falling has detrimental effects on older patients' wellbeing. Falls can lead to fractures, brain injuries, healthcare utilization, increasing healthcare cost, and loss of independency [6–8, 17]. In addition, postfall syndrome includes intense fear about falling and mobility disorders after fall event [8, 18]. Fear of falling may lead to significant psychological consequences such as loss of confidence and independency as well as activity avoidance [8]. Moreover, it has been shown that falls are associated with lower health-related quality of life (HRQOL) in older adults with cancer after controlling for demographic, health, and cancer-related factors [19]. The high prevalence of falling among older patients with cancer who undergo surgery indicates the significance of evaluating patients' fall history in preoperative assessments and finding its associations with other geriatric syndromes. Another finding was the significantly higher occurrence of falls among women than men. The higher risk of falls in women could be attributable to the higher mean age in this group as compared with men group.

Our study showed that our patients were more likely to experience their most recent fall at home rather than outside the home. While we could not find any other study assessing this finding in older patients with cancer, prior studies on community-dwelling older adult populations have shown that most falls, especially in frail people, happen at home [20, 21]. This is likely due to older frail patients spending more time at home. The above finding emphasizes the need for effective interventions in preventing further falls in the postoperative setting, such as home safety interventions and occupational therapists' home visits [22, 23].

In the present study, we assessed the relationship between the functional status, as measured by ADL, IADL, and KPS score, of preoperative older patients with cancer and absence and presence of fall and number of falls in the prior year. For all of these functional status assessments, we observed significant associations between history of falling and functional status. Our findings show that the percentage of patients with KPS score of 80 or less doubled from no fall group to patients with one or more falls during past year. The strong association between KPS scores and falls also found in similar studies has caused some investigators to suggest this measure as a useful independent tool for identifying patients at risk for falls [24].

To assess mobility among preoperative older patients with cancer we measured their timed get up and go. We noticed that the longest TUG test time (20 seconds or more) was most frequently seen among patients with multiple falls history. TUG test has been used in combination with other measures to predict falls in older patients [25]. This is consistent with our observation that worse TUG test results are associated with more falls.

We also observed significant differences in all items of IADL between these three groups. The percentage of patients being unable to do an instrumental daily activity rose sharply from those with no falls group to those with one and more than one falls. In IADL questions, the highest rates of disability among patients with multiple falls were observed in housekeeping and shopping. Another study assessing the association of falls with daily activity profile in geriatric patients also has found the greatest differences between fallers and nonfallers in shopping and light household work [26]. Shopping is a complex activity requiring several physical, mental, and social skills. Older patients with fall history are very often unable to do their shopping because of functional decline, history of fall [27], and their fear of falling [8]. Housekeeping disability can also be explained by the same factors. Inability to keep the home clean in older patients with cancer who live alone leads to an increased risk of falling due to an unsafe environment [28]. Failing to perform these two activities independently, especially in the postoperative setting, may lead to more dependency, and if the further needs are not met, this may lead to subsequent adverse outcomes (e.g., falls and hospital readmission). This can further emphasize the need to screen older patients with cancer undergoing surgery for the history of fall. Those with history of falling may be frailer than those without history of falling. Involvement of a social worker and case manager in the postoperative setting may assist in further exploration of the needs and dependencies of older adults with cancer.

Our study confirmed that, among ADL items, walking outside was the most frequently limited activity, followed by bladder/bowel control and bathing. Almost one-third of patients with a history of multiple falls were very limited in walking outside home. These patients were more confined to home and as a result suffered more falls at home rather than outside (data not shown). The difficulty with bladder/bowel control can be clinically relevant to fall because of medication use such as alpha blockers used for incontinency treatment [29]. As a result, reviewing and modifying patients' medication list in the postoperative setting is important for preventing further falls. Bathing, on the other hand, is a high-risk activity due to the hazardous environment involving a wet and slippery surface for older people who are prone to falling [28]. Increased deficit in this activity after one or multiple falls can be caused by postfall syndrome [8, 27] leading to psychological and physical limitation after fall. Existing fall prevention interventions including exercise, physical therapist and occupational therapist visits, home safety measures, and multifactorial strategy are effective in reducing the fall events in older patients with cancer [30, 31].

We also analyzed the use of walking assistive devices among older adults with cancer. We noticed a high proportion of patients from either group that were not using any devices. Although the rate of using assistive devices was highest among those with multiple falls compared to the two other groups, still half of patients with multiple falls were not using any devices. This is despite the fact that walking assistive devices when used correctly are of benefit in fall prevention [23, 30]. A recent study by Mettelinge et al. revealed an increased risk of future falls with relation to use of assistive devices. It was explained by an altered spatiotemporal gait pattern, increased age, and psychotropic drug intake [32]. Also, using unsuitable walking aids as well as lack of proper training periods could be other potential reasons for increased risk of falls [32, 33]. Hence, the medical professionals should select the most fitted device according to their patients' needs and provide them with the necessary education and instructions [33].

In the current study, we noticed a high proportion of patients from either group that were not using any devices. In a study on community-dwelling older adults with fall history, the most frequent reasons for nonuse of canes and devices included believing it was not needed, forgetfulness, the device making them feel old, and inaccessibility [34]. The same study found that nonuse led to a significantly higher risk of falls resulting in surgery than among device users [34]. The underlying reasons for underutilization of these devices in this population should be investigated in the future studies.

In summary, our findings in preoperative older oncology population indicate the importance of assessing fall history before cancer surgery. Studies have shown that one of the strongest predictors of fall in the future is occurrence of fall in the past [35, 36]. Moreover, older adults with cancer are at higher risk compared to younger patients for developing postoperative complications, prolonged hospital stay, and functional decline [37, 38]. A recent study examining hospital records of older patients undergoing major cancer surgeries has shown that at least one geriatric event (i.e., dehydration, delirium, fall, and failure to thrive) occurs in 9.2% of these cases after surgery [39]. Of these events, 9.6% were mobility-related (pressure ulcers, falls, and fractures) [39]. While the study did not assess the correlation between mobility-related events and prior history of fall, as mentioned above, those with prior history of falls could be at higher risk for these events. Thus, patients with history of falls need special interventions (e.g., physical therapy assessment) in the postoperative period to reduce the risk of future falls.

We had a large cohort of patients and very limited missing information which further strengthened our study. However, given the single institution study, the findings may not be generalizable to older patients with cancer undergoing surgery elsewhere. Another limitation for our study is including only patients who were referred to geriatrics service. It is possible that more frail patients were referred to geriatrics service and as a result the prevalence of fall among all older patients with cancer undergoing surgery is less than what our study shows. However, it is critical to ask about history of falling when assessing older adults with cancer in the surgical clinic. Inquiring about falls can also be done in other preoperative evaluations performed by others (e.g., primary care provider). Our research mainly aimed to find out a relationship between functional status or use of assistive devices and falls. Therefore, some important risk factors for falls such as medications, orthostatic hypotension, poor vision, behavioral factors, and environmental hazards were not evaluated. Another limitation to our study is the possibility of underreporting bias caused by poor memory in older patients. It is possible that patients with one-time fall did not recall their fall and as a result selected no fall in answering the question. If this is true, the actual prevalence of fall among these patients would be higher than we reported and hence another reason to screen patients for history of fall. In our study we were not able to determine a temporal sequence. Did older patients first develop geriatric syndromes and then fell, or did they fall and as a result of sustaining injuries develop more geriatric syndromes? The relationship between frailty and geriatric syndromes could turn into a vicious cycle where one leads to another, which in turn increases the risk of the first event. A multidimensional approach toward geriatric syndromes and fall is needed to break this vicious cycle. This causal relationship between fall and geriatrics syndromes needs to be explored in future prospective study by following patients postoperatively.

## 6. Conclusion

Based on our findings and prior knowledge, we conclude that falls are prevalent among older adults with cancer presenting for preoperative evaluation. They are associated with geriatric syndromes and outcomes. Further attention should be paid to both primary and secondary prevention since patient outcomes deteriorate as each fall happens. Health providers should be more attentive to mobility needs of older patients with cancer and consider more frequent evaluations of their cancer patients' needs for walking assistive devices. Further studies are needed to assess the impact of fall history on surgical recovery and outcomes of older patients with cancer.

## Figures and Tables

**Table 1:** Sociodemographic characteristics of patients.

	Fall −	Fall +	Total	*p* value
	(72.4%)	(26.7%)		
Age				
Less than 80	48.4%	44.5%	47.7%	0.079
80 or more	51.6%	55.5%	52.3%	
Sex				
Female	46.7%	56.4%	49.5%	0.025
Male	53.3%	43.6%	50.5%	
Marital status				
Married	54.8%	47.7%	53.0%	0.685
Nonmarried	45.2%	52.3%	47.0%	
Household type				
Living alone	31.2%	31.5%	31.3%	0.371
Not living alone	68.8%	69.5%	68.7%	

## Data Availability

The datasets generated during and analyzed during the current study are available from the corresponding author on reasonable request.
